# A *Coxiella* mutualist symbiont is essential to the development of *Rhipicephalus microplus*

**DOI:** 10.1038/s41598-017-17309-x

**Published:** 2017-12-14

**Authors:** Melina Garcia Guizzo, Luís Fernando Parizi, Rodrigo Dutra Nunes, Renata Schama, Rodolpho M. Albano, Lucas Tirloni, Daiane Patrícia Oldiges, Ricardo Pilz Vieira, Wanderson Henrique Cruz Oliveira, Milane de Souza Leite, Sergio A. Gonzales, Marisa Farber, Orlando Martins, Itabajara da Silva Vaz, Pedro L. Oliveira

**Affiliations:** 1 Instituto de Bioquímica Médica Leopoldo de Meis, Universidade Federal do Rio de Janeiro, Rio de Janeiro, RJ Brazil; 2 Centro de Biotecnologia, Universidade Federal do Rio Grande do Sul, Porto Alegre, RS Brazil; 3 Laboratório de Biologia Computacional e Sistemas, IOC - Fiocruz, Rio de Janeiro, RJ Brazil; 4 Departamento de Bioquímica, IBRAG, Universidade do Estado do Rio de Janeiro, Rio de Janeiro, RJ Brazil; 5 Faculdade de Veterinária, Universidade Federal do Rio Grande do Sul, Porto Alegre, RS Brazil; 6 Departamento de Química, Universidade Federal Rural do Rio de Janeiro, Seropédica, RJ Brazil; 7 Instituto Nacional de Ciência e Tecnologia em Entomologia Molecular (INCT-EM), Rio de Janeiro, RJ Brazil; 8 Instituto de Biotecnologia, Centro de Investigaciones en Ciencias Veterinarias y Agronómicas, INTA, Buenos Aires, Argentina

## Abstract

The cattle tick *Rhipicephalus microplus* is a hematophagous ectoparasite that causes important economic losses in livestock. Different species of ticks harbor a symbiont bacterium of the genus *Coxiella*. It was showed that a *Coxiella* endosymbiont from *R*. *microplus* (CERM) is a vertically transmitted mutualist symbiont, comprising 98% of the 16S rRNA sequences in both eggs and larvae. Sequencing of the bacterial genome revealed genes for biosynthetic pathways for several vitamins and key metabolic cofactors that may provide a nutritional complement to the tick host. The CERM was abundant in ovary and Malpighian tubule of fully engorged female. Tetracycline treatment of either the tick or the vertebrate host reduced levels of bacteria in progeny in 74% for eggs and 90% for larvae without major impact neither on the reproductive fitness of the adult female or on embryo development. However, CERM proved to be essential for the tick to reach the adult life stage, as under antibiotic treatment no tick was able to progress beyond the metanymph stage. Data presented here suggest that interference in the symbiotic CERM-*R*. *microplus* relationship may be useful to the development of alternative control methods, highlighting the interdependence between ticks and their endosymbionts.

## Introduction

The recent explosive growth of microbiome investigations have revealed an unsuspected dimension of the metazoan-associated microbial world, establishing the understanding of symbiotic interactions and their impact on host physiology as central issues in integrative biology. Mutualistic symbionts can provide nutrients for the host, defense against pathogens or natural enemies, and insecticide resistance^1–4^. Furthermore, the interaction with their invertebrate hosts has emerged as a potential target for arthropod control^5,6^.

Ticks are blood-sucking parasites of humans, pets, livestock and wildlife. They harbor a wide variety of microorganisms, such as bacteria, viruses and protozoa^7,8^. The cattle tick *Rhipicephalus* (*Boophilus*) *microplus* is responsible for large economic losses in livestock due to skin damage, decreases in animal weight gain and milk production, and transmission of pathogens^9^. Since the end of the 19^th^ century, tick control has relied on acaricides^10^, which have lost efficiency due to the spread of resistance, justifying the search for alternative control methods^11,12^.

*R*. *microplus* is a one-host tick, presenting a free-living stage and a parasitic stage that lives on the host body. The ectoparasite feeds and molts twice on the same host (from larva to nymph and from nymph to adult) in a period that takes about three weeks. After mating, the adult female starts the slow feeding phase, when tick swallows moderate amounts of blood (about 5 days). The rapid engorgement phase takes about 2 days where a massive amount of blood is ingested^13^. After completing the blood meal, the fully engorged female detaches from the cattle host and performs the oviposition in the soil. Finished egg laying, the female dies and the hatched larva parasites a new host.

Several tick species have been shown to harbor pathogenic and non-pathogenic bacteria that are closely related to the *Coxiella* genus. The non-pathogenic bacterium (referred to as *Coxiella* endosymbiont, CE) were identified in ticks from the genera *Rhipicephalus*, *Amblyomma*, *Haemaphysalis*, *Ornithodoros*, *Argas* and *Carios* ^14–20^, being the most common maternally transmitted symbiont in ticks^21^. In some species, however, other bacterial species, such as *Midichloria* and *Francisella*, apparently have successfully replaced the CE^21^. Zhong *et al*.^22^ showed that treatment with antibiotics eliminated the CE from *Amblyomma americanum*, and compromised reproductive success by reducing hatching of the larvae. A genomic analysis of this symbiont showed that it forms a monophyletic clade with the pathogenic bacterium *Coxiella burnetii* ^23^. Despite sharing a common ancestor, very large genomic differences exist between these two organisms, with the symbiont showing a highly-reduced genome, which is less than one-third the size of the genome of *C*. *burnetii*. This work also revealed that the CE from *A*. *americanum* is a potential source of B complex vitamins and cofactors^24^.

Here, it was investigated the interaction between a CE and *R*. *microplus*. Tetracycline treatment of either the tick or the vertebrate host showed that *Coxiella* sp. is a mutualistic vertically transmitted symbiont that is essential to tick physiology. *Coxiella* endosymbiont from *R*. *microplus* (CERM) is crucial for the maturation of the metanymph to the adult stage, which indicated that the interaction between the CERM and *R*. *microplus* has a role in tick physiology that is distinct from conclusions of previous studies on CE-tick relationship. It is suggested that disruption of the symbiotic relationship between the CE and *R*. *microplus* is a target that may be used in the development of alternative control methods for this ectoparasite.

## Results

### A CERM is the predominant bacterium in *R*. *microplus* egg and larva

**Figure 1:**
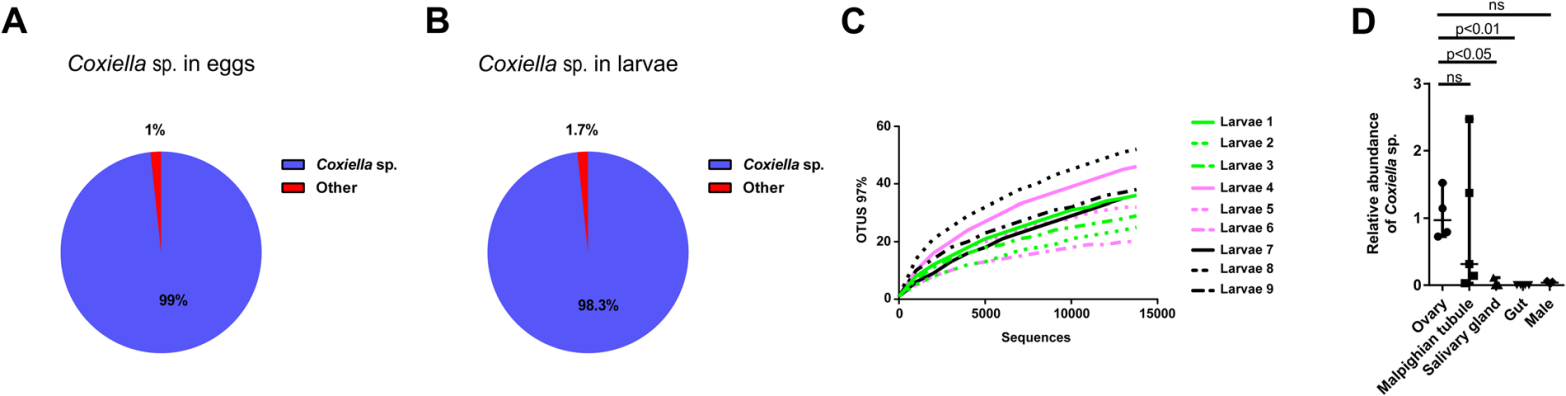
CERM in *R*. *microplus*. (**A**) Bacterial genera in *R*. *microplus* eggs; (**B**) Bacterial genera in *R*. *microplus* larvae. The “Other” group includes *Enterobacter* (0.4%), *Nocardiopsis* (0.4%) and *Rubrobacter* (0.2%). Genera with frequencies below 0.1% are not represented; (**C**) Rarefaction curve of the 16S rRNA gene sequences in larvae based on OTUs determined at 97% similarity; (**D**) Quantification of CERM in ovary, Malpighian tubule, salivary gland and gut of fully engorged females by qPCR. The adult male was also analyzed. The levels of CERM were expressed as the median with 95% of CI of five biological samples with two technical replicates in each point. Statistical analyses: Kruskal-Wallis test for (**D**). ns: not statistically significant.

A 16S rRNA survey performed with *R*. *microplus* eggs revealed that ninety-five sequences out of ninety-six clones sequenced were identical and corresponded to a bacterium belonging to the genus *Coxiella* (Fig. 1A). To identify the bacteria found in newly hatched *R*. *microplus* larvae, a 16S rRNA survey was performed using Illumina technology, which showed that a single OTU (operational taxonomic unit) classified as a *Coxiella* sp. accounted for 98.3% of the reads from larval samples (Fig. 1B). The rarefaction curve (Fig. 1C) confirmed that sequencing coverage was sufficiently extensive to fully evaluate the microbial diversity. Therefore, these results reveal that the *Coxiella* species found in *R*. *microplus* eggs and larvae (CERM) is a vertically transmitted symbiont.

### CERM is abundant in ovary and Malpighian tubule of fully engorged female *R*. *microplus*

The abundance of CERM in the adult male, ovary, salivary gland, gut and Malpighian tubule of fully engorged females was analyzed by qPCR (Fig. 1D). The CERM was present in all tissues analyzed, but the relative abundance was higher in ovary of fully engorged females, followed by Malpighian tubule. Low levels were detected in salivary gland of adult females, but the presence of the CERM in the saliva of fully engorged females was not detectable by PCR. Very low levels were found in adult males and gut of fully engorged females.

### Genomic analysis of CERM

The genome sequencing showed an apparently complete CERM genome by comparison with other *Coxiellas* genomes (CRt – *Coxiella* symbiont of *Rhipicephalus turanicus*, CLEAA – *Coxiella* symbiont of *Amblyomma americanum* and *C*. *burnetii* RSA 493) (Table S1). A list of CERM genes identified here possibly involved in the interaction with *R*. *microplus* is shown in Table S2, revealing the presence of biosynthetic pathways for several vitamins and key metabolic cofactors. As exceptions are the biosynthetic pathway for Thiamine (B1), which is highly degraded in CERM and CRt, while a complete set of enzymes is found in CLEAA, and the pathway for Nicotinate (B3), which is not found in CERM and CRt, but present in CLEAA. The genome from CERM, as well as the other *Coxiellas* the also coded for genes involved in nitrogen metabolism (Table S3), such as those for the synthesis of essential amino acids phenylalanine, tryptophan, threonine, valine, leucine and isoleucine.

### Levels of the CERM were affected in fully engorged females injected with tetracycline

**Figure 2:**
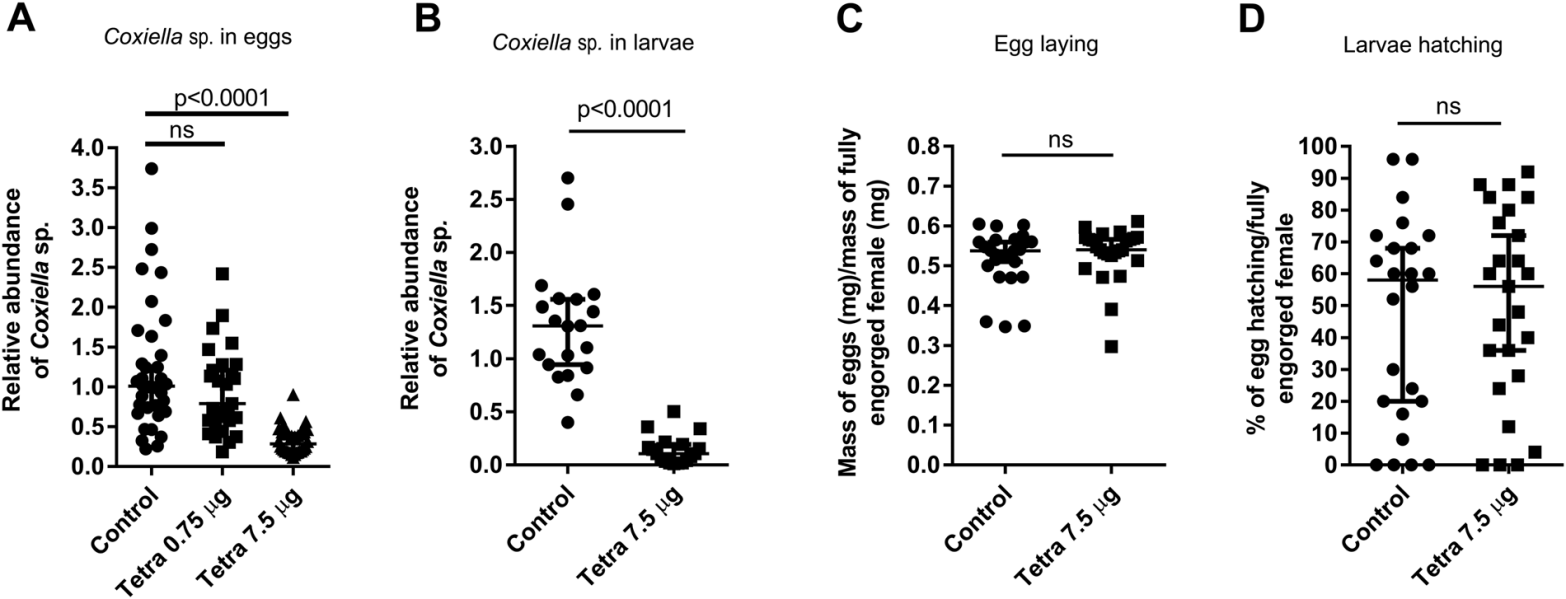
Reproductive fitness of fully engorged females injected with tetracycline and levels of CERM in eggs and larvae. (**A**) CERM in eggs by qPCR; (**B**) CERM in larvae by qPCR; (**C**) Oviposition index; D- Percentage of hatching. The levels of CERM were expressed as the median with 95% of CI of a biological triplicate with two technical replicates to each point. Statistical analyses: Kruskal-Wallis test for (**A)**. Mann Whitney test for (**B**–**D**). ns: not statistically significant. The effect size calculated by Cohen’s d method was 2.9 (89%), −0.16 (−4%) and −0.13 (−8%) for (**B**–**D**), respectively.

Tetracycline in a dose of 0.75 µg/tick was not able to reduce the quantity of the CERM in the eggs (Fig. 2A). However, a higher dose (7.5 µg/tick) effectively reduced the levels of the bacterium in the eggs (Fig. 2A) and in the larvae (Fig. 2B) but did not affect oviposition or egg hatching (Fig. 2C and D).

### The development of larva with reduced levels of the CERM was arrested at the metanymph stage

**Figure 3:**
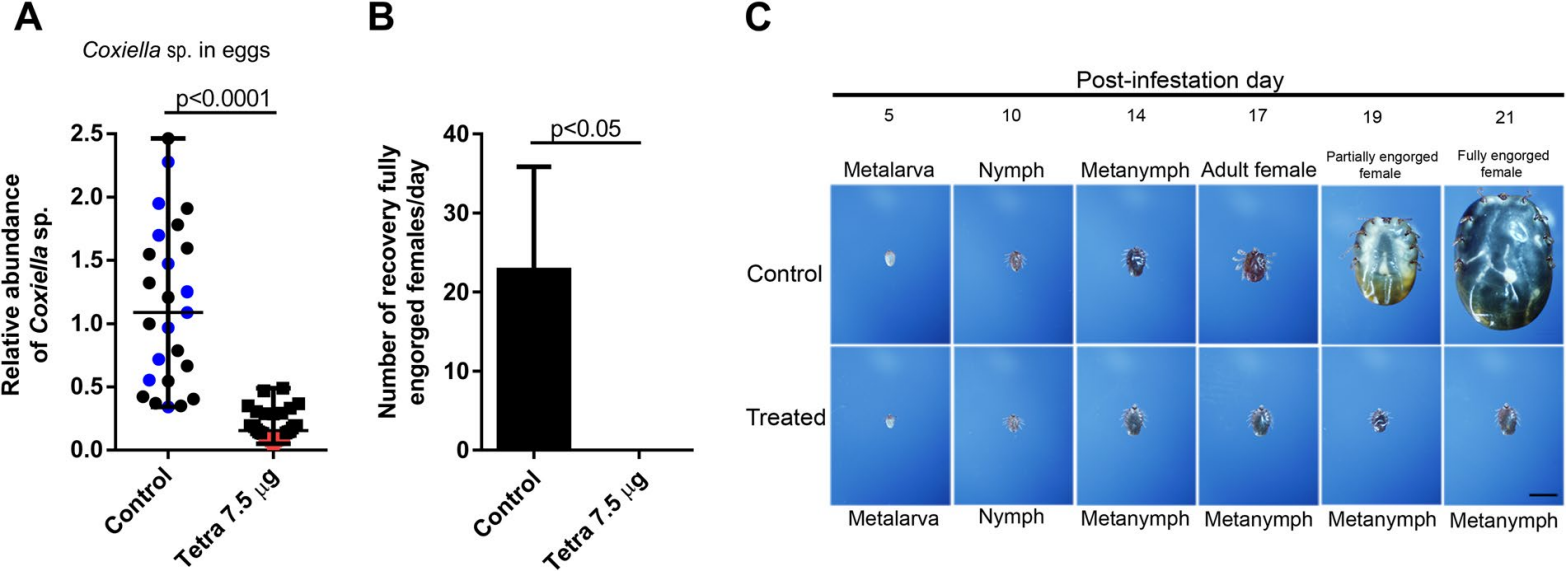
Effect of tetracycline treatment of the tick on the development of the progeny of *R*. *microplus*: (**A**) Levels of CERM in eggs of fully engorged females injected with tetracycline. The selected batches of eggs whose hatched larvae were used for cattle infestation in control and treated groups are indicated in blue and red symbols, respectively; (**B**) Fully engorged females recovered at the 21^st^, 22^nd^, 23^rd^ and 24^th^ days post-infestation from the control and treated groups after infestation with 1 gram of 10-day-old larvae; (**C**) Phenotype of progeny collected on different days post-infestation. Scale bar: 2 mm. The levels of CERM are expressed as the median with 95% of CI of a biological triplicate with two technical replicates to each batch of eggs. Statistical analyses: Mann Whitney test for (**A** and **B**). The effect size calculated by Cohen’s d method was 2.3 (83%) for (**A**).

The relative abundance of the CERM was reduced in eggs of tetracycline-treated fully engorged females compared with that in the eggs of non-treated ticks (Fig. 3A). One gram of eggs was chosen among the clutches of eggs presenting the most pronounced decrease in CERM levels after tetracycline administration (indicated by the red circles in Fig. 3A). A control group of eggs was chosen from eggs laid by control females from the same experiment (indicated by the blue circles). One head of cattle was seeded simultaneously with larvae hatched from tetracycline-treated and non-treated eggs. Each larva group was allowed to feed in a delimited space by closed cotton bags glued to the animal’s skin as described in the Methods. *R*. *microplus* development was followed through the collection of ticks at different post-infestation days (Fig. 3B and C). Ticks of both groups showed a similar time course of development until the 14^th^ day, at the metanymph stage. After this day, metanymphs from the tetracycline-treated group did not develop further, whereas the non-treated ticks went through the regular developmental profile: appearance of the metanymphs at the 14^th^ day and adult males and females on the 17^th^ day, followed by full engorgement of the females by the 21^st^ day (Fig. 3C). After the 14^th^ day, only metanymphs were recovered in the tetracycline-treated group. These metanymphs could attach to the host, but were not able to mature further and did not molt into the adult life stage.

### The progeny of fully engorged females fed on tetracycline-treated cattle blocked development at the metanymph stage

**Figure 4:**
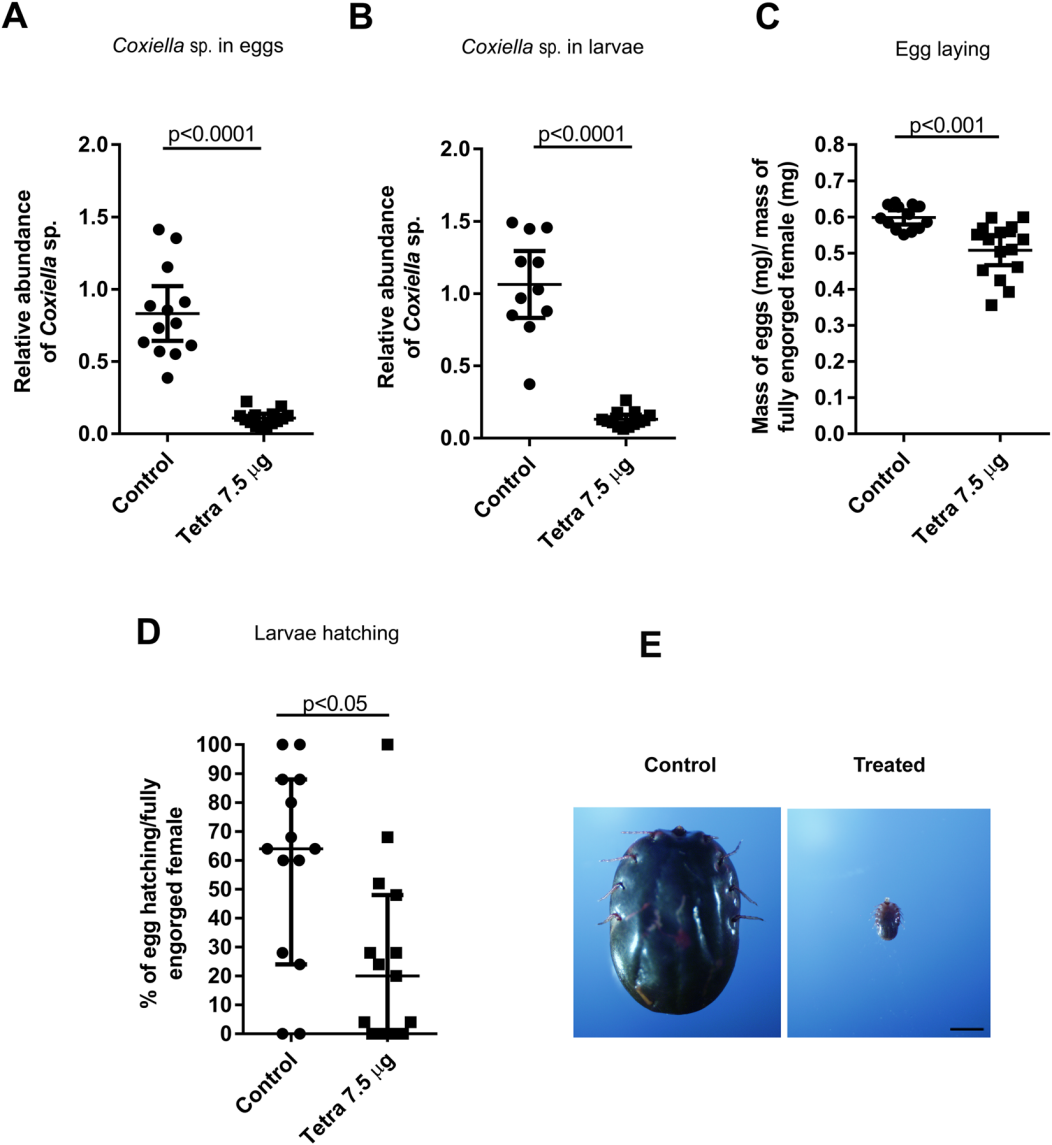
Levels of CERM and reproductive fitness of fully engorged females fed on cattle treated with tetracycline. (**A**) CERM levels in eggs by qPCR; (**B**) CERM levels in larvae by qPCR; (**C**) Oviposition index; (**D**) Percentage of hatching; (**E**) Phenotype of progeny from treated and non-treated females collected on 21^st^ day post-infestation. Scale bar: 2 mm. The levels of CERM were expressed as the mean with 95% of CI for (**A**) (**B** and **C**) and as the median with 95% of CI for (**D**) of a biological triplicate with two technical replicates to each point. Statistical analyses: t test for (**A**–**C**) and Mann Whitney test for (**D**). The effect size calculated by Cohen’s d method was 3.4 (87%), 3.1 (87%), 2 (15%) and 1.1 (57%) for (**A**–**D**), respectively.

The results obtained with ticks injected with tetracycline, led us to hypothesize that if ticks fed on tetracycline-treated cattle, which is a situation more closely associated with the typical tick physiology, a similar effect would be achieved. One head of cattle was intramuscularly injected with three doses of 12 grams of tetracycline, applied at 12-hour intervals. This dosage was based on the treatment of fully engorged females at a dose of 7.5 µg/tick. Antibiotic levels found in both cattle blood (10.7 and 16.3 ng/ml) and in the gut of fully engorged females (7.2 and 20.3 ng/ml) were measured by mass spectrometry, confirming that effective concentrations were reached. Levels of the CERM were markedly reduced in the eggs and the larvae (Fig. 4A and B) from tick females fed on tetracycline-treated cattle. In contrast to the results obtained with the injection of the antibiotic into the tick, oviposition and hatching were also significantly reduced after treatment (Fig. 4C and D). The capacity of the larvae to develop on the host was also evaluated. One pool of larvae (hatched from one gram of eggs laid by 15 fully engorged females that were fed on tetracycline-treated cattle) was seeded onto one head of an untreated cattle. After 21 post-infestation days, the ticks were collected, and only metanymphs were found in the pool taken from the tetracycline-treated cattle, which agrees with the results shown in Fig. 4C and E). As a control, a head of an untreated cattle was seeded with larvae hatched from eggs laid by non-treated females, which, as expected, completed their life cycle on the 21^st^ post-infestation day, when fully engorged females were recovered (Fig. 4E).

## Discussion

Although it is generally accepted that most arthropods are infected with heritable bacteria, in most cases the physiological role of these bacteria on their host’s biology has not been determined^25^. Maternally transmitted CE bacteria were identified in different tick species^8,16,20,26,27^. Here, using a 16S rRNA survey, we identified a CE in the cattle tick *R*. *microplus* as a dominant bacterium in eggs and larvae. One previous communication reported that *R*. *microplus* did not have a CE^28^, but the failure to detect this bacterium was probably due to the use of an PCR reaction based on a CE from *Amblyomma cajennense*. The bacterium found in our study is from the same genus found in *R*. *microplus* ovary by Andreotti *et al*.^19^, who speculated that the bacterium could be maternally transmitted, which has been experimentally demonstrated here and also by Duron *et al*.^23^.

Arthropods that feed exclusively on vertebrate host blood subsist in an unbalance diet, that although extremely rich in some nutrients, such as iron and amino acids, has a very low concentration of essential micronutrients, such as vitamins^29,30^. Symbionts can provide a nutritional complement to hosts as showed to the obligate hematophagous. Tsetse fly harbor the bacterium *Wigglesworthia glossinidia* that supplies vitamins that are lacking in the strictly hematophagous diet of the insect^31^. The presence in the CERM genome of a full complement of enzymes needed to synthesize several vitamins and enzyme cofactors supports the hypothesis that *R*. *microplus* obtain these molecules from the endosymbiont, as suggested for the *Coxiella* symbiont of *A*. *americanum* (CLEAA) and *R*. *turanicus* (CRt)^24,32^. The biosynthetic pathways for the vitamins Biotin (B7), Riboflavin (B2), Pyridoxine (B6), Folic acid (B9) and Pantothenate (B5) were found in all analyzed CEs. The Thiamine (B1) pathway is present is CLEAA but is highly degraded in CERM and CRt, while the Nicotinate (B3) is present in CLEAA but is not found in CERM and CRt. The coding genes for the cofactors Flavin adenine dinucleotide (FAD) and Coenzyme A (CoA) were found in all analyzed CLEs, while the pathway for biosynthesis of Nicotinamide adenine dinucleotide phosphate (NADP^+^) is degraded in the genomes of CERM and CRt, but present in CLEAA.

An early event in the evolution of metazoans was the loss of most of essential genes from the biosynthetic pathways for essential amino acids^33^. Some bacteria are able to provide essential amino acids to their hosts, as showed for the whitefly mutualistic symbiont, *Candidatus Portiera aleyrodidarum* ^34^. CERM genome also encodes genes involved in the synthesis of essential amino acids. Ticks feed large volumes of blood and the high protein content of vertebrate blood in a first glance makes it seems unlikely that providing additional amino acids would be of any physiological relevance for this ectoparasite. However, the initial developmental stages of the tick, the larva, metalarva, nymph and metanymph feed much lower volumes of blood than the following stages, and the data presented here indicate that the critical contribution of CERM occurs at some point before the tick starts to ingest massive amounts of blood. Therefore, an interesting possibility that deserves further investigation is that providing an extra supply of essential amino acids in early developmental stages might be one of the contributions of the CEs to the tick host.

Clearance of the bacteria from an arthropod is a common approach to interfere in host-symbiont interactions when testing for effects on host physiology. Tetracycline administration, either by injection into the tick hemocoel or through treatment of the vertebrate host decreased the CERM levels and demonstrated the mutualistic character of this symbiotic association. Both treatments resulted in the interruption of tick development at the metanymph stage, indicating a critical contribution by the bacterium shortly before the metanymph molting to the adult life stage. Although both treatments had a similar effect in the reduction of CERM levels, only the females that were fed on treated cattle showed a significant inhibition of oviposition and hatching. It is possible that the exact moment when the females were exposed to tetracycline dose has affected the results. Ticks fed on treated cattle received antibiotic in an earlier life stage when comparing with the tick injection that were performed in the fully engorged female. Thus, it is plausible that different aspects of the female physiology may be affected not just by number of CERM, but also by the moment when bacteria were eliminated. Different from our findings, in *A*. *americanum*, a lower but comparable dose of tetracycline injected in the hemocoel led to a decrease in oviposition and hatching^22^. However, the decrease in CE levels attained in that report was more pronounced than that obtained here. Therefore, it is possible that CERM has a different physiological role in *R*. *microplus* when compared to CE from *A*. *americanum*, or that the observed effects are dependent on the amount of bacteria remaining after antibiotic treatment. It is known that tetracycline acts through binding to the bacterial ribosome inhibiting protein synthesis. However, at high doses the antibiotic can also be toxic to animal cells. Although it was not possible to completely eliminate the CERM, it is likely that the interruption of the tick development was due to the reduced level of CERM and not to a toxic effect of tetracycline on tick cells, because the effects of the antibiotic were observed not in the adult female submitted to the treatment, but in its progeny.

As tissue distribution of the symbiont might be indicative of its role in the biology of the arthropod host, we investigated its levels in *R*. *microplus*. In our study, the CERM was identified in males as well as in several organs of fully engorged females. It was also found in egg and larva, showing the occurrence of transovarial transmission. The identification of a CERM in ovary is in accordance with other reports of heritable symbionts, where colonization of the reproductive tissue was shown to allow infection of the offspring^2^. Among all organs, ovary presented the higher levels of CERM, as has been shown in *R*. *turanicus* ^35^. The CERM was also identified in high levels in Malpighian tubule, which is in accordance with Lalzar *et al*.^27^ that showed the presence of a *Coxiella* sp. in *R*. *turanicus* and Klyachko *et al*.^36^ that found a bacterium from the same genus in *A*. *americanum* Malpighian tubule. The identification of the bacterium in Malpighian tubule suggest a role of CERM in the metabolism of nitrogen. The presence in salivary gland is also in agreement with the findings reported for other tick species^28,36,37^. The *Ixodes ricinus* symbiont *Midichloria mitochondrii* was found in the saliva, and antibodies against the bacteria were found in the sera of humans and animals exposed to tick bites^38^. However, although the CERM was found in the salivary gland, through the methodology used in this study it was able to show that the bacterium was not present in saliva of fully engorged female. The presence of bacterium in the salivary gland also raises the possibility that another physiological role for the CERM could be related to the development of the salivary gland, which might explain the blockage at the metanymph stage since tick saliva contains molecules with anti-inflammatory, immunosuppressive and anti-hemostatic properties that are essential for blood feeding^39^. Very low levels of CERM were identified in adult male, reinforcing the hypothesis of maternal transmission of CERM.

The reduction of the CERM in egg and larva of fully engorged females fed on tetracycline-treated cattle showed that the antibiotic circulating in the blood was able to cross the tick’s gut barrier and reach the hemolymph. The commercial use of antibiotics to control parasites is probably not feasible due to environmental and public health concerns, as an expected outcome would be the selection of resistant bacterial strains^40^. However, the comprehension of the role of the CERM in *R*. *microplus* biology could lead to alternative control methods that directly target the symbiont or tick proteins involved in the interaction with the bacterium^8^. Furthermore, blocking tick development at this early stage prevents most of the damage to cattle caused by massive blood loss that occur after a rapid engorgement phase that follows the metanymph stage, probably also limiting the transmission of pathogens to the next generations. Several groups are pursuing a vaccine against *R*. *microplus* using tick proteins as antigens and high levels of protection of the host against ticks have been achieved^12,41^. The description of the genome of CERM showed that the bacterium is a vitamin and cofactor provider, as well as other CEs. Description of the CERM genome together with the genomes of CE from *A*. *americanum* and *R*. *turanicus* provides useful information for understanding how *Coxiella* sp. bacteria interact with their hosts^24,32^. In this juncture, the data provided here suggest that the microbiota or tick molecules involved in the interaction with the mutualistic symbiont could be considered as a promising targets to vaccine development^42^.

## Methods

### *R. microplus* strain

*R*. *microplus* ticks (Porto Alegre strain) were maintained under laboratory conditions, through in Hereford cattle obtained from a naturally tick-free area^43,44^. The colony was kept isolated, without the introduction of wild ticks. All animals were housed in individual tick-proof pens on slatted floors. During the experiments, fully engorged females, eggs and larvae were kept under laboratory conditions in an incubator at 28 °C and 80% relative humidity. All animal care and experimental protocols were conducted following the guidelines of the institutional care and use committee (Ethics Committee on Animal Experimentation of the Federal University of Rio Grande do Sul) and were approved under the registry 14403/protocol 07.

### 16S rRNA survey and analysis from *R*. *microplus* eggs

A pool of 30 mg of newly laid eggs from *R*. *microplus* had the surface sterilized with 70% ethanol, followed by a washing with sterile water. The sample was homogenized in PBS followed by gDNA extraction and library construction^45^. Ninety-six clones from the 16S rRNA library were sequenced by capillary electrophoresis on a MegaBace 1000 DNA analysis system (GE Healthcare, Little Chalfont, UK). The partial 16S rRNA sequences (880-bp) were analyzed with the BLAST tool against the GenBank database. The partial CERM 16S rRNA gene sequence was deposited in GenBank under the accession number KT726373.

### 16S rRNA survey and analysis from *R*. *microplus* larvae

Nine pools of larvae hatched from 30 mg of eggs laid by three fully engorged females each were washed once in 70% ethanol and twice in sterile water. The larvae were homogenized in sterile water, and DNA was extracted from 100 µl of the homogenate with the Power Soil DNA Isolation Kit (MO BIO, Carlsbad, CA, EUA). PCR was performed with the AccuPrime Taq DNA Polymerase High Fidelity (Invitrogen, Carlsbad, CA, EUA) using primers targeted to the V4 region of the 16S rRNA gene in a dual-index strategy^46^. The amplicons were purified with PureLink Quick Gel Extraction & PCR Purification Combo kit Fidelity (Invitrogen, Carlsbad, CA, EUA). Equimolar concentrations of the nine purified PCR products were sequenced on an Illumina MiSeq platform. Fastq paired-end reads were assembled at the RDP pipeline website (version 11. 4) using their extended version of PANDAseq with a minimum read Q score of 25, sequence length between 240 and 275 bp and no ambiguities as parameters^47^. The assembled amplicon sequences were checked for chimeras using the UCHIME tool^48^ on the Fungene pipeline of the RDP website. After assembly and the first chimera check, samples were processed with Mothur following the MiSeq SOP^46,49^. Sequences were aligned to the SILVA bacterial 16S rRNA reference alignment, and those that did not align were discarded^50^. Unique sequences were identified and a preclustering step was performed to further reduce the sequencing errors obtained from clustering sequences with up to two nucleotide differences^51^. The resulting sequences were screened for chimeras using UCHIME. Sequences were then classified against the Mothur-formatted version of the RDP 16S rRNA gene training set (version 9) with a confidence score of 80%. Sequences that classified as Archea, Eukarya, chloroplast or mitochondria were excluded from further analysis. OTUs were assigned at a 3% dissimilarity level, and richness estimates were performed.

### Genome sequencing and analysis of CERM

Genomic DNA was extracted from five ovaries of fully engorged *R*.*microplus* adult females, using DNeasy Blood & Tissue Kit from Quiagen (32.9 ng/uL) (Hilden, NRW, DE) and sequenced using the Illumina MiSeq platform (Paired-end Nextera XT library). The data comprised nearly 8 × 10^6^ paired-end reads of 500 bp. Total raw reads were subjected to trimming using Trimmomatic version 0.33^52^ and filtered after mapping against the complete genome of *Coxiella* endosymbiont of *R*. *turanicus* (CRt - NZ_CP011126) and *Coxiella* endosymbiont of *A*. *americanum* (CLEAA - NZ_CP007541.1) using Bowtie 2 (version 2.2.3)^53^ to obtain a smaller set of 99232 paired-end reads. Flash (version 1.2.11)^54^ was used to merge pairs of mapped raw reads. The short sequence patterns in genomic data analysis (k-mer analysis) revealed a genome coverage of nearly 32X. Celera Assembler version 8.3rc2 was used for *de novo* assembly^55^ followed by the SPAdes genome assembler version 3.5.0 using the Celera resulting output as trusted contigs^56^. Further, a gap filling step (IMAGE tool from suite PAGIT, version 2.4)^57^ was performed using the whole set of original reads (8 × 10^6^ paired-end reads), resulting into 215 contigs, after the elimination of less than 200 bps length ones. Additionally, to get rid of potential tick contamination, BLASTN (version 2.2.26+)^58^ was used for local alignment against *Rhipicephalus microplus* (LYUQ01000000). Finally, 171 contigs were obtained, which were submitted to NCBI GenBank (SRR5860283). The completeness of the assembled genome was inspected using a single-copy gene database^59^, and by comparison with CRt, CLEAA and *C*. *burnetii* RSA 493 (NC_002971.4), using the same single-copy gene analysis. The curated contigs were annotated using the RAST Server^60–62^.

### Tetracycline treatment of ticks

Groups of 10 fully engorged females (average 250 mg/tick) were washed once in 70% ethanol and twice in sterile water. Tetracycline hydrochloride (Merck, Darmstadt, HE, DEU) was administered in sterile 137 mM NaCl, 2.7 mM KCl, 4.3 mM sodium phosphate, and 1.4 mM potassium phosphate, pH 7.0 (PBS) at concentrations of 0.75 µg/µl and 7.5 µg/µl, based on the treatment of Q-fever in humans (30 mg/kg/day)^63^. The injection (1 µl of tetracycline at concentrations described above or PBS in control group) was performed with a micro-syringe (Hamilton - 33-gauge needle) into the hemocoel by the ventral surface of females in the pre-oviposition period on the 2^nd^ day after natural detachment. The needle was left for one minute inside the tick before removal, allowing the antibiotic to be distributed through the hemolymph.

### Vertebrate host treatment

On the 19^th^ and 20^th^ days post-infestation with 1 gram of ten-day old larvae, the cattle (approximately 400 kg) received intramuscular applications of three 12 gram doses of a veterinary use solution of 100 mg/ml tetracycline hydrochloride - Solutetra (Ibasa, Porto Alegre, RS, BRA), administered at intervals of 12 hours. The concentration of tetracycline used in the cattle was based on the treatment of fully engorged females at a concentration of 7.5 µg/µl.

### Tetracycline quantification in cattle blood and the gut of fully engorged females

In the treatment of cattle with tetracycline, the levels of the antibiotic in the cattle blood and in the gut of fully engorged females that were fed on the animal were quantified by tandem mass spectrometry. EDTA was added to the samples and the samples were extracted with acetonitrile. Two samples of cattle blood were collected: one 4 hours after the first administration of the antibiotic and the other at the end of the treatment. In addition, two guts were collected after the natural detachment of the fully engorged females at the end of the cattle treatment.

### Sample collection for RNA isolation and cDNA synthesis

After tetracycline treatment, fully engorged females were kept in an incubator until oviposition completion. The eggs laid on the 1^st^ day were weighed and collected to quantify the levels of CERM. After five days of embryonic development, the eggs were macerated in TRIzol reagent (Invitrogen, Carlsbad, CA, EUA) for RNA extraction. The eggs laid on the 2^nd^ day were weighed and maintained at incubation until hatching. Larvae at the 10^th^ day after hatching were further macerated to quantify the levels of CERM.

Fully engorged females (230–300 mg; average 250 mg/tick) and adult males were collected after natural detachment or were manually removed from the cattle, respectively. Tick female dissections were performed under a microscope in sterile PBS. After the removal of the dorsal cuticle with dissecting scissors, the specific organs were isolated with fine-tipped forceps. Ovary, Malpighian tubule, gut and the salivary gland of fully engorged females were macerated in TRIzol (Invitrogen, Carlsbad, CA, EUA). Each ovary, Malpighian tubule and gut sample contained two organs each, whereas salivary gland samples represented a pool of four organs. Each male sample contained a single individual.

RNA concentration and purity of all samples were determined on a NanoDrop spectrophotometer. The cDNA was synthesized from equal amounts (1 µg) of total RNA using the High Capacity cDNA Reverse Transcription kit (Applied Biosystems, Waltham, MA, EUA).

### Saliva collection for gDNA extraction

Saliva was collected from 30 fully engorged females after administration of 5 µl of 2% pilocarpine (Merck, Darmstadt, HE, DEU) in PBS on the ventral surface. The sample was collected in Tris-EDTA, pH 8.0 and loaded with 10 µg of salmon sperm DNA (Sigma-Aldrich, St. Louis, MO, EUA). gDNA extraction was performed using the phenol-chloroform method^64^ and its concentration and purity were determined using a NanoDrop spectrophotometer.

### CERM relative abundance analysis

The CERM relative abundance was measured in samples with a primer pair (forward-5′TTCGGTGGGAAAGAAAGTTTC3′; reverse-5′TAGGGCTTTCACATTCGACTTAAAT3′) specific for the 16S rRNA gene sequence (KT726373) of the CERM that was identified in 16S rRNA survey from eggs. *R*. *microplus* 40 S ribosomal gene (EW679928) was used as a reference gene for data normalization after amplification with specific primers (forward-5′GGACGACCGATGGCTACCT3′; reverse 5′TGAGTTGATTGGCGCACTTCT3′)^65^. The identity of the amplicons was confirmed by Sanger sequencing. qPCR was carried out on a 7500 Real-Time PCR System (Applied Biosystems, Waltham, MA, EUA) with 40 cycles of 95 °C (15 s) and 60 °C (45 s) following an initial denaturation of 95 °C (10 min). A melting curve was generated to confirm the identity of the amplicons. Each 15 µl reaction mixture contained 7.5 µl of 1x Power SYBR Green PCR Master Mix (Applied Biosystems, Waltham, MA, EUA), 3.5 µM of each primer, and 5 µl of cDNA 10x diluted in sterile water. Relative abundance was analyzed by the comparative Ct method^66^.

### Analysis of the presence of CERM

The detection of the CERM in the saliva of fully engorged females was performed by PCR. Each 15 µl reaction mixture contained 7.5 µl of PCR Master Mix (Fermentas, Waltham, MA, EUA), 10 µM of the CERM 16S rRNA gene primers and 1 µl of gDNA in sterile water. The gDNA of salivary glands of fully engorged females was used as a positive control. The reaction was carried out in a 96-well thermal cycler (Applied Biosystems, Waltham, MA, EUA) with 35 cycles of 95 °C (30 s), 60 °C (30 s) and 72 °C (30 s) with an initial denaturation of 95 °C (5 min) and a final incubation of 70 °C (10 min). The amplicon (178 bp) was visualized using 1.5% agarose gel electrophoresis.

### Evaluation of the effect of antibiotic on reproductive fitness

The ratio between the weight of the pre-oviposition fully engorged females and the total mass of the eggs laid after tetracycline treatment was used as an oviposition index. To determine the percentage of egg hatching, 25 eggs from each fully engorged female were randomly selected following oviposition completion. After egg hatching, the larvae and infertile eggs were frozen and counted under a microscope.

### Effect of antibiotic on larvae viability

Ten-day-old larvae hatched from eggs of tetracycline-treated and non-treated tick females were used to simultaneously infest a head of cattle through two cloth bags glued to animal skin on the lumbar region^67^. Ticks were collected at the 5^th^, 10^th^, 14^th^, 17^th^, 19 ^th^, 21^st^, 22^nd^, 23^rd^ and 24^th^ days post-infestation and fixed with 4% paraformaldehyde in 100 mM cacodylate buffer, pH 7.4. Tick life stages were determined using a microscope, and images were acquired with an Olympus DP72 digital camera.

Ten-day-old larvae hatched from one gram of eggs were used to infest one tetracycline-treated and one untreated head of cattle, and fully engorged females were collected after natural detachment. Subsequently, the larvae obtained from the eggs laid by these females were used to infest new, untreated cattle. On the 21^st^ day post-infestation, ticks from both cattle were collected, fixed and examined as described above.

### Statistical analysis

All the data were tested for normality using Shapiro-Wallis test. The differences among three or more groups were determined using Kruskal-Wallis test. Other comparisons were performed using Mann Whitney test or two-tailed t-test depending on gaussian distribution. The values were statistically significant when p < 0.05. All statistical calculations were performed using GraphPad Prism software (version 7) except the effect size, which was calculated as the Cohen’s d^68^.

### Data Availability

The partial 16S rRNA gene sequences from larvae were deposited in GenBank under the accession codes SRR2681092, SRR2681676, SRR2681677, SRR2681678, SRR2681680, SRR2681682, SRR2681683, SRR2681684 and SRR2681685. The partial CERM 16S rRNA gene sequence from eggs was deposited in GenBank under the accession number KT726373. The genome of CERM were deposited in GenBank under the accession code NSHJ01000000.

## Electronic supplementary material

Supplemental file
