# Supplementary material for: A Coxiella mutualist symbiont is essential to the development of Rhipicephalus microplus
Source: Sci Rep. 2017 Dec 14;7:17554. doi: 10.1038/s41598-017-17309-x (PMC5730597; doi:10.1038/s41598-017-17309-x)
Supplement: Supplementary file 1 — Supplemental file [file 41598_2017_17309_MOESM1_ESM.pdf]

Title:

A *Coxiella* mutualist symbiont is essential to the development of *Rhipicephalus microplus*

Authors:

Melina Garcia Guizzo[1], Luís Fernando Parizi[2], Rodrigo Dutra Nunes[1], Renata Schama[3], Rodolpho M. Albano[4], Lucas Tirloni[2], Daiane Patrícia Oldiges[2], Ricardo Pilz Vieira[1], Wanderson Henrique Cruz Oliveira[6], Milane de Souza Leite[6], Sergio A. Gonzales[8], Marisa Farber[8], Orlando Martins[1], Itabajara da Silva Vaz Junior[2,5,7], Pedro L. Oliveira[1,7*]

Feature	CERM	CRt	CLEAA	Cb
Genome Size (Mbp)	1.19	1.7	0.66	1.99
GC (%)	32.66	38.2	34.6	42.6
Protein coding (%)	56.4	48.5	83.9	89.1
Genes	836	912	551	2,094
Single copy genes	104/111	102/111	101/111	106/111

Table S1: Genomes of *Coxiella* endosymbionts of ticks. CERM – *Coxiella* endosymbiont from *R. microplus*; CRt – *Coxiella* endosymbiont from *R. turanicus*; *Coxiella* endosymbiont from *A. americanum*; Cb – *Coxiella burnetii* RSA 493.

Biosynthetic pathway	CERM	CRt	CLEAA
**Vitamins**			
**Biotin (B7)**			
bioC	CLERM_746	WP_048875477.1	WP_084588734.1
bioH	CLERM_627	WP_048875315.1	WP_039670095
bioF	CLERM_628	WP_048875316.1	WP_039670096
bioA	CLERM_630	WP_048875318	WP_039670098.1
bioD	CLERM_625	WP_048875796.1	WP_052246377.1
bioB	CLERM_629	WP_048875317.1	WP_039670097.1
**Riboflavin (B2)**			
ribA	CLERM_180	WP_048875046.1	WP_039669871.1
ribD	CLERM_586	WP_048875044.1	WP_039669869.1

ribH	CLERM_181	WP_048875047.1	WP_039669872.1
ribE	CLERM_179	WP_048875045.1	WP_039669870.1
**Pyridoxine (B6)**			
GAPDH	CLERM_412	WP_048874822.1	WP_039669757.1
pdxB	CLERM_445	WP_048874810.1	WP_039669763
serC	CLERM_670	WP_048875157.1	WP_039670132.1
pdxA	CLERM_535	WP_048875681.1	WP_039669901.1
pdxJ	CLERM_654	WP_048874958.1	WP_039669818.1
pdxH	CLERM_431	WP_048875231.1	WP_039670071.1
**Folic Acid (B9)**			
folE	CLERM_473	WP_048875401.1	WP_039670042.1
folB	CLERM_275	WP_048875676.1	WP_039669906.1
folK	CLERM_084	WP_048875541.1	WP_052246374.1
folP	CLERM_217	WP_048875417.1	WP_039670037.1
folC	CLERM_566	WP_048875798.1	WP_039670066.1
folA	CLERM_231	WP_048875685.1	WP_052246324.1
**Pantothenate (B5)**			
panB	CLERM_765	WP_048875491.1	WP_039670235.1
panC	CLERM_766	WP_048875492.1	WP_039670003.1
**Thiamine (B1)**			
thiC	**Absent**	**Absent**	WP_052246335.1
thicDE	**Absent**	**Absent**	WP_084588732.1
nifS	CLERM_515	WP_048875792.1	WP_039670083.1
ThiF	**Absent**	**Absent**	WP_039669760.1
thifG	**Absent**	**Absent**	WP_039669989.1
**Nicotinate (B3)**			
nadB	**Absent**	**Absent**	WP_039669917.1
nadA	**Absent**	**Absent**	WP_039670112.1
nadC	**Absent**	**Absent**	WP_039669915.1
**Cofactors**			
**Flavin adenine dinucleotide (FAD)**			
ribF	CLERM_259	WP_048875815.1	WP_052246375.1
**Coenzyme A (CoA)**			
coaA	CLERM_270	WP_048875600.1	WP_052246330.1
coaBC	CLERM_569	WP_048875349.1	WP_039670064.1
coaD	CLERM_083	WP_048875540.1	WP_039669974.1
coaE	CLERM_091	WP_048875620.1	WP_039669930.1
**Nicotinamide adenine dinucleotide phosphate (NADP+)**			
nadB	**Absent**	**Absent**	WP_039669917.1
nadA	**Absent**	**Absent**	WP_039670112.1
nadC	**Absent**	**Absent**	WP_039669915.1
nadD	CLERM_522	WP_048875184.1	WP_039670121.1
nadE	CLERM_583	WP_048875362.1	WP_039670054.1
ppnK	CLERM_641	WP_048875461.1	WP_039670015.1

Table S2: Biosynthetic pathways for the synthesis of vitamins and cofactors in *Coxiella* endosymbionts genomes. CERM – *Coxiella* endosymbiont from *R. microplus*; CRt – *Coxiella* endosymbiont from *R. turanicus*; CLEAA - *Coxiella* endosymbiont from *A. americanum.*

Biosynthetic pathway	CERM	CRt	CLEAA
**Biosynthesis of essential aminoacids**			
**Phenylalanine and tryptophan**			
pyridoxal phosphate-dependent aminotransferase	CLERM_664	WP_048875152	WP_084588741.1
5-Enolpyruvylshikimate-3-phosphate synthase	CLERM_803	WP_048875158.1	WP_039670281.1
3-dehydroquinate dehydratase I	CLERM_806	WP_048875721.1	WP_039670147.1
2-keto-3-deoxy-D-arabino-heptulosonate-7-phosphate synthase I alpha	CLERM_133	WP_048875304.1	WP_039670078.1
Chorismate synthase	CLERM_574	WP_048875353.1	WP_039670062.1
Shikimate 5-dehydrogenase I alpha	CLERM_662	WP_048875740.1	WP_039669745.1
3-dehydroquinate synthase	CLERM_063	WP_048874781.1	WP_039669746.1
Shikimate kinase I	CLERM_064	WP_082160486.1	WP_052246297.1
**Threonine**			
Phosphoserine aminotransferase	CLERM_670	WP_048875157.1	WP_039670132.1
2-amino-3-ketobutyrate coenzyme A ligase	CLERM_836	WP_048875640.1	**Absent**
Serine hydroxymethyltransferase	CLERM_416	WP_048875001.1	WP_039669841.1
**Valine, leucine and isoleucine**			
Leucine dehydrogenase	CLERM_280	WP_048875043.1	WP_039670196.1
Dihydrolipoamide acyltransferase component of branched-chain alpha-keto acid dehydrogenase complex	CLERM_653	WP_048875040.1	WP_039669867.1
Branched-chain alpha-keto acid dehydrogenase, E1 component, alpha subunit	CLERM_651	WP_048875042.1	WP_084588725.1

Table S3: Enzymes involved in the synthesis of different essential amino acids in *Coxiella* endosymbionts genomes. CERM – *Coxiella* endosymbiont from *R. microplus*;

CRt – *Coxiella* endosymbiont strain from *R. turanicus*; *Coxiella* endosymbiont of *A. americanum.*
